# Long-term effects of motherfit group therapy in pre-(MOTHERFIT1) and post-partum women (MOTHERFIT2) with stress urinary incontinence compared to care-as-usual: study protocol of two multi-centred, randomised controlled trials

**DOI:** 10.1186/s13063-019-3331-6

**Published:** 2019-04-25

**Authors:** Heidi F. A. Moossdorff-Steinhauser, Esther M. J. Bols, Marc E. A. Spaanderman, Carmen D. Dirksen, Mirjam Weemhoff, Fred H. M. Nieman, Bary Berghmans

**Affiliations:** 1 Faculty of Health, Medicine and Life Sciences, Department Epidemiology, CAPHRI Care and Public Health Research Institute, Maastricht University, P.O. Box 616, 6200 MD Maastricht, The Netherlands; 2 Maastricht University Medical Centre, P.O. Box 5800, 6202 AZ Maastricht, The Netherlands; 3 Department of Obstetrics and Gynecology, Maastricht, The Netherlands; 4 Pelvic Care Center Maastricht, Maastricht, The Netherlands; 5 Department of Clinical Epidemiology and Medical Technology Assessment, Maastricht, The Netherlands; 6 Department of Obstetrics and Gynecology, Zuyderland Medisch Centrum, P.O. Box 5500, 6130 MB Sittard-Geleen, The Netherlands

**Keywords:** Cost-effective, Group therapy, Motherfit, Pelvic-floor-muscle training, Peri-partum, Post-partum, Pregnancy, Pre-partum, Randomised controlled trial, Stress urinary incontinence

## Abstract

**Background:**

Stress urinary incontinence (SUI) is highly prevalent during pregnancy and after delivery. It is often associated with a failing pelvic floor, sphincteric and/or supportive system. Pelvic-floor-muscle training (PFMT) peri-partum has been proven effective for up to 1 year post-partum; however, its long-term effects are unknown. Group PFMT, given by a physiotherapist, has been proven to be as equally effective as individual therapy. Motherfit is a group-PFMT therapy with an emphasis on pelvic floor exercises, adherence and general fitness. Care-as-usual (CAU), if guideline driven, should, as first treatment option, consist of PFMT. Cost-effective strategies are of relevance, given the rise of health care costs. Motherfit group therapy has the potential to be cost-effective in women with urinary incontinence. Therefore, the objectives of the two current studies are: (1) to investigate whether intensive, supervised, pre-partum (MOTHERFIT1) or post-partum (MOTHERFIT2) pelvic-floor-muscle group therapy reduces 18-month post-partum severity of SUI compared to CAU and (2) whether MOTHERFIT1 OR MOTHERFIT 2 is more (cost-)effective compared to CAU.

**Methods:**

Two multi-centred, randomised controlled trials (MOTHERFIT1, *n* = 150, MOTHERFIT2, *n* = 90) will be performed. Participants will be recruited by their midwife or gynaecologist during their routine check. Participants with SUI will receive either motherfit group therapy or CAU. Motherfit group therapy consists of eight group sessions of 60 min each, instructed and supervised by a registered pelvic physiotherapist. Motherfit group therapy includes instructions on pelvic floor anatomy and how to contract, relax and train the pelvic-floor muscles correctly and is combined with general physical exercises. Adherence during and after motherfit will be stimulated by reinforcement techniques and a mobile app. The primary outcome measure is the absence of self-reported SUI based on the severity sum score of the International Consultation on Incontinence Questionnaire Short Form (ICIQ-UI-SF) at 18 months post-partum. Secondary outcomes evaluate quality of life, subjective improvement and health care costs.

**Discussion:**

The motherfit studies are, to our knowledge, the first studies that evaluate both long-term results and health care costs compared to CAU in pregnant and post-partum women with SUI. If motherfit is shown to be (cost-)effective, implementation in peri-partum care should be considered.

**Trial registration:**

Netherlands Trial Register, ID: NL5816. Registered on 18 July 2016.

**Electronic supplementary material:**

The online version of this article (10.1186/s13063-019-3331-6) contains supplementary material, which is available to authorized users.

## Background

Urinary incontinence (UI) affects 13–40% of women during their life [1–4]. Pregnancy and childbirth are the most important provocative factors for UI during lifetime [5].

Stress urinary incontinence (SUI), defined as any involuntary leakage of urine on effort or exertion, or on sneezing or coughing, is the most prevalent type of UI during pregnancy [6]. SUI can be the result of a failing pelvic floor, sphincteric and/or supportive system [7]. The prevalence of SUI rises from approximately 9% in the first trimester of pregnancy to 32% in the second and 38% in the third trimester [8–10]. Eight weeks after delivery the prevalence of SUI is 19%, rising to, respectively, 22% and 26% at 6 and 12 months post-partum [8, 11]. Mørkved et al. [10] even reported a prevalence of 40% at 8 weeks post-partum. Many women believe that their UI will resolve by itself [12]. However, it is known that 75 to 92% of the women with SUI at3 months post-partum, still have UI even after 5 or 12 years [13, 14]. Often, UI reduces quality of life (QoL) because of its negative impact on sexual relationships and daily life activities [15, 16]. Despite this, 75% of women never seek help for UI because they feel embarrassed or feel that losing urine is normal after giving birth [12, 17, 18].

Pelvic-floor-muscle training (PFMT) aims to improve the supportive system and to achieve a timely contraction in case of expected leakage, both with voluntary (the Knack manoeuvre) and involuntary contractions [19]. Positive effects of PFMT peri-partum are proven up to 1 year post-partum [20]. However, it is still unknown whether the long-term effects are lasting as well as whether pre- or post-partum PFMT is more effective in treating SUI compared to care-as-usual (CAU). Currently there are no guidelines on UI peri-partum for midwifes and gynaecologists [21]. Therefore, CAU is known to be applied differently among health care providers and sometimes only includes prescription of incontinence materials [22]. PFMT may be provided individually or in a group. Recently, a meta-analysis on the effects of individual versus group PFMT for women with UI, both provided by a physiotherapist, showed no significant difference in results between the groups [23]. The latter is of particular interest as group therapy is less expensive when compared to individual therapy, and might, therefore, be a cost-effective strategy. It is known that health care costs are rising due to an increasing level of unhealthy lifestyle and number of people with one or more chronic diseases. For that reason, it is of relevance to focus on the evaluation of potentially cost-effective therapies [24, 25].

Given the promising effects of PFMT in the short term and the potential of group therapy being a cost-effective strategy, the Pelvic care Center Maastricht (PcCM), embedded in the Maastricht University Medical Centre (MUMC+), developed motherfit group therapy. Motherfit group therapy includes pelvic-floor-muscle group therapy (PFMGT) combined with general fitness exercises, provided by pelvic physiotherapists (PPTs), to treat peri-partum women with SUI. Moreover, motherfit group therapy has a strong focus on self-management, as it is reported that this will promote adherence and thereby sustain longer-term effects [26].

The primary objective of this study is to investigate whether a structured assessment and treatment programme (motherfit group therapy) of intensive, supervised PFMGT, including a home maintenance programme, reduces 18 months’ post-partum UI severity (frequency, amount and impact) compared to CAU in adult pregnant women (MOTHERFIT1) and post-partum women with SUI (MOTHERFIT2). The secondary objective is to investigate whether motherfit group therapy is cost-effective compared to CAU in pregnant (MOTHERFIT1) and post-partum women with SUI (MOTHERFIT2) 18 months post-partum.

It is hypothesised that intensive, supervised, pre-partum (study 1: MOTHERFIT1) or post-partum (study 2: MOTHERFIT2) PFMGT is more (cost-)effective compared to CAU in adult pregnant (MOTHERFIT1) or post-partum women with SUI (MOTHERFIT2).

## Methods

### Study design

The study consists of two multi-centred, randomised controlled trials (RCTs), namely MOTHERFIT1 and MOTHERFIT2. MOTHERFIT1 focusses on investigating PFMGT pre-partum and MOTHERFIT2 on PFMGT post-partum. Participants will be recruited in the southern part of The Netherlands from the Maastricht University Medical Center (MUMC+), Zuyderland MC (Heerlen/Sittard), Laurentius Hospital (Roermond), Maxima MC (Eindhoven) and surrounding midwifery practices. Except for Maxima MC, all obstetric centres are part of the Obstetric Consortium Limburg, a first-, second- and third-line obstetric midwifery maternity care collaboration. In every region, a registered PPT will provide motherfit group therapy. This protocol has been prepared in accordance with standard protocol items; recommendation for interventional trials (SPIRIT), a completed SPIRIT checklist is included as Additional file 1.

### Participants

Women will be included if they meet all of the following criteria: (1) aged ≥ 18 years, (2) UI (stress or mixed with predominant stress UI factor, according to Haylen et al. [6], (3) a score of > 3 on the International Consultation on Incontinence Modular Questionnaire-Urinary Incontinence-Short Form (ICIQ-UI-SF) questionnaire [27], (4) are motivated for participation in the motherfit programme, (5) are competent to speak and understand the Dutch language and to read and fill in forms independently and (6) have a mobile app (mApp) on a tablet (Apple or Android) available.

Exclusion criteria are: (1) UI prior to first pregnancy, still existing during pregnancy, (2) high-risk pregnancy, resulting in a contra-indication for performing intensive pelvic-floor-muscle (PFM) exercises (e.g. placenta praevia, vaginal blood loss, preterm uterine contractions), (3) suffering from significant exercise limitations or co-morbidities (physical or psychological) that would restrain a woman from participation in motherfit group therapy, (4) a history of chronic neurological disorders or diseases related to UI (e.g. multiple sclerosis, cerebrovascular accident, diabetes mellitus (during ≥ 1 year with glycated hemoglobin (HbA1c) > 10 mmol/l)), (5) urinary tract infection (urine-sediment, urine culture), (6) a history of anti-incontinence or urogynaecological surgery, (7) women who are expected to be lost to follow-up (e.g. because of a planned change of residency), (8) have had recent pelvic physiotherapy (< 6 months) and (9) refusal to use a mApp.

### Detailed study plan

#### Patient recruitment/consent procedure

The obstetrician/gynaecologist or midwife (case manager) at each centre will be responsible for identifying eligible participants. All women will receive written and digital (www.motherfit.net) general information about the motherfit study at:

- the first visit to the case manager and may be recruited from the second visit at 12 weeks or later until 27 weeks’ gestation (MOTHERFIT1)
- routine control at 6 weeks post-partum (MOTHERFIT2)

**Fig. 1:**
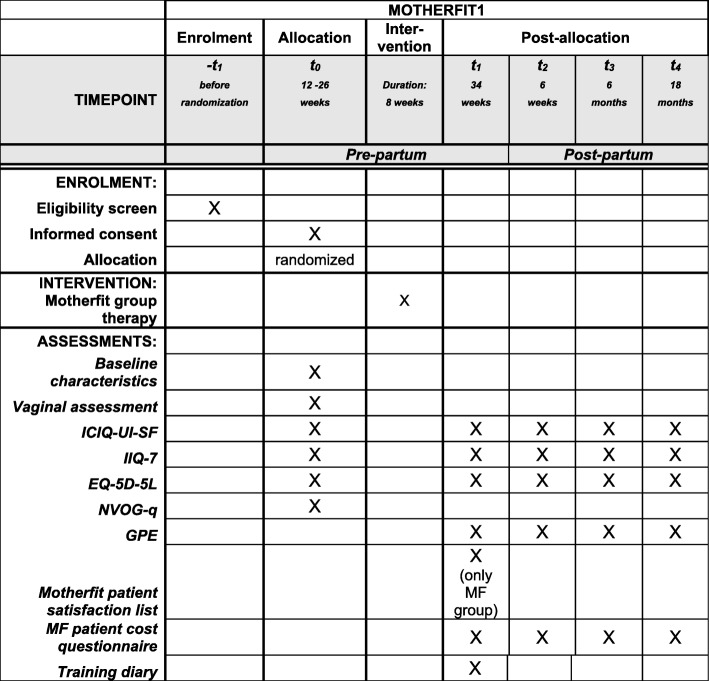
Schedule of enrolment, allocation, interventions and assessments for MOTHERFIT1

**Fig. 2:**
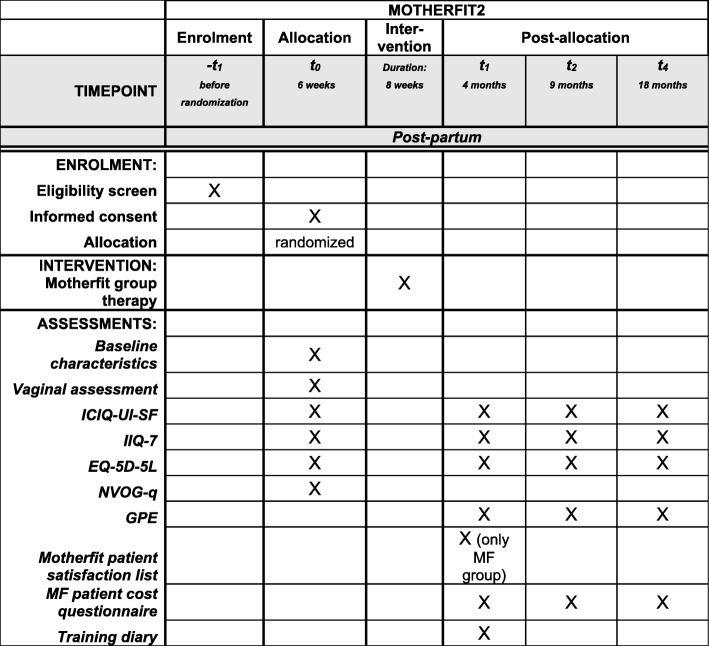
Schedule of enrolment, allocation, interventions and assessments for MOTHERFIT2

In case a woman is interested to participate, a simple vaginal examination is performed to check the ability to contract the pelvic floor muscles (Figs. 1 and 2). The woman will receive an envelope containing: patient information, two informed consent forms with return envelope and an information booklet on the medical scientific research of the Dutch Government [28]. The case manager fills in the name, telephone number and email address of the woman at a secure site (digital database), which can only be accessed by the researcher. After 1 week, the researcher will contact the woman by telephone and ask whether she has any questions regarding the study after reading the patient information. If the woman is willing to participate, she will be asked to fill in the two informed consent forms and return them to the researcher. The researcher will sign the two informed consent forms and return one to the participant.

### Allocation of participants

After signing the informed consent, the participant will receive an email with a link to the electronic baseline questionnaires. Once the questionnaires are completed, block randomisation (block size is 4) will be done by a computer-generated sequence in a 1:1 ratio on the individual patient and location level. Allocation in blocks of 4 is concealed and done using a central computer. Participants are either allocated to the motherfit programme (intervention) or the CAU (control group).

### Blinding

Due to the nature of the interventions, the participants and PPTs cannot be blinded. During the trial the coordinating researcher is not blinded. However, once the participant has completed the questionnaires, it is not possible to make changes in the data due to locking of the questionnaires. Moreover, before the statistical analyses all participants will be appointed a new study number for which the coordinating researcher is blinded. Therefore, analyses will be done blinded for treatment allocation.

### Protocol training

#### Case managers

Preceding the inclusion period, on-site information, instruction on the standardised assessment and inclusion procedures will be provided to case managers by the researcher for 1 h. Assessment follows the standard procedures of the Dutch Consortium Urogynecology to assess pelvic floor signs and symptoms. Special attention will be paid to the short assessment of a correct contraction of the PFMs by observation and vaginal palpation for closing and lifting of the PFMs [29].

### Pelvic physiotherapists (PPTs)

In The Netherlands, pelvic physiotherapy is a specialisation within the field of physiotherapy and has its own registration in order to guarantee quality [30].

Preceding the inclusion period, information, instruction and training on the standardised assessment and group therapy protocol will be provided to involved PPTs during a 2-h workshop. The PPTs receive a set of laminated A4 papers with a detailed description for each therapy session, containing: topics to discuss, PFM and homework exercises.

### Interventions

#### Care-as-usual

In case participants with SUI are allocated to the CAU group, participating case managers give their normal advice and women make their own choices as to whether they want to participate in any kind of pregnancy-related course, visit to a physician or therapist.

#### Motherfit group therapy

**Fig. 3:**
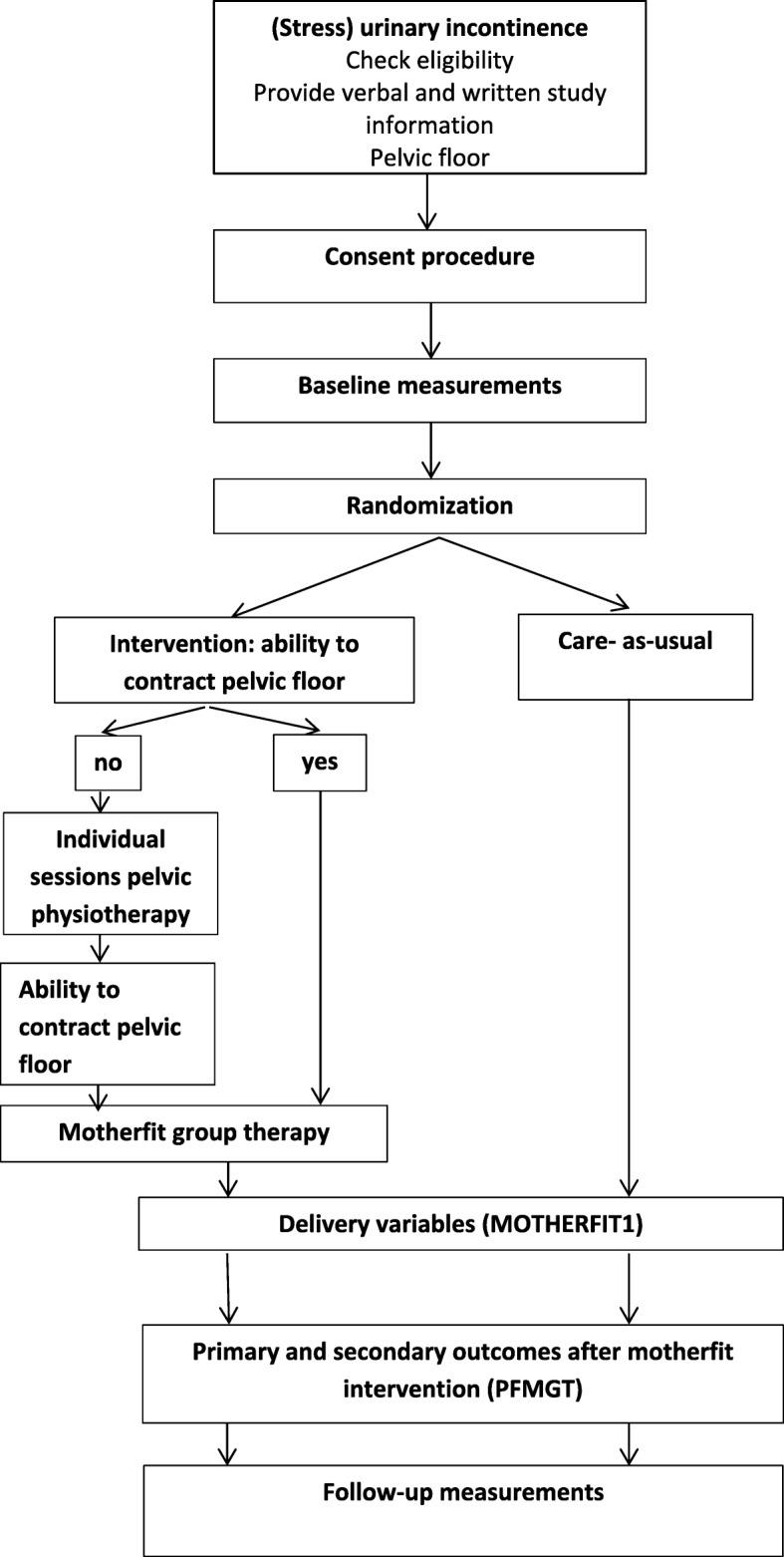
Standard Protocol Items: Recommendations for Interventional Trials (SPIRIT) Figure. *PFMGT* pelvic-floor-muscle group therapy

All women allocated to motherfit group therapy, and unaware or unable to contract their PFMs correctly, will be referred to the PPT for individual instruction before joining the motherfit group therapy (Fig. 3). Every participating region has a PPT who provides individual or group therapy. Motherfit consists of eight group therapy sessions of 60 min each, instructed and supervised by a registered PPT. In each group a maximum of four women are allowed to participate. Women of both studies can start when they have been randomised to motherfit group therapy. Therefore, the participant’s group composition may change over time. Motherfit includes instructions on pelvic floor anatomy and how to contract, relax and train the PFMs correctly and is combined with general physical exercises with a strong focus on self-management.

The PFMT protocol has been published previously by Bø et al. [31], and is based on the Norwegian Aerobic Fitness Model. It follows the general training principles and the recommendations concerning physical activity practice during and after pregnancy according to the American College of Obstetricians and Gynecologists and the World Health Organisation (WHO) [31, 32] (Table 1).

**Table 1:** Types of training provided during MOTHERFIT1 and MOTHERFIT2 with accompanying aim and exercises

Type of training	Aim	Exercise(s)
Awareness	Continue breathing during PFM contraction	Breathing and PFM exercises
Skills	Consciously timed voluntary pre-contraction	The ‘Knack’– closing of vaginal hiatus and in-, up- and forward movement of the PFMs before and during increased abdominal pressure
Functional	Increase awareness to avoid unnecessary abdominal pressure and to prevent unnecessary or extreme perineal descent during daily activities	Correct pushing technique during defecation, or a PFM contraction in situations associated with a rise in abdominal pressure
Muscle strength and endurance	Build up long-lasting muscle volume, providing structural support/‘stiffness’, resulting in reduced perineal descent	Slow velocity • Build up to 8–12 contractions, of 6–8 s (if possible), add 3–4 fast contractions on top at the end to recruit more slow-twitch fibres. Start with double time rest (complete relaxation) between contractions • Three sets of exercises during the day in varying positions: lying, sitting, kneeling, standing position • Preferably daily training, but minimally 3–4 days a week, during at least 5–6 months • Maintenance muscle strength after 6 months’ training; 2 days a week where intensity is more important than frequency
Muscle contraction: speed	Build up explosive strength	Fast repetitions • Build up from 10 sets of 3 quick contractions to 10 sets of 5 quick contractions, 3 times a day

*PFM* pelvic-floor-muscle training

Moreover, all women receive a mApp (iPelvis) [33], an application with individualised pelvic physiotherapy exercises, and supportive video content and images. Performance and adherence to PFMT will be recorded in the participants’ personal training diary and is reinforced by the regularly sending of push notifications on the mApp. The training diary will be available for the motherfit group therapists and may be used to discuss the participants’ motivation to incorporate adequate PFMT and use of PFM in their daily activities. Although adverse events due to PFMT are very rare [20], adverse event forms are used to register their occurrence during the motherfit group therapy.

### Data collection and outcome measures

All data (electronic case report forms and questionnaires at baseline and follow-up) of the participants and case managers will be collected in a (web-based) digital central database. Demographic variables and personal characteristics will be registered by the Nederlandse Vereniging voor Obstetrie & Gynaecologie vragenlijst (NVOG-q) at baseline for MOTHERFIT1 and MOTHERFIT2.

MOTHERFIT1: data will be collected at baseline, 34 weeks of gestation, 6 weeks and 6 and 18 months post-partum.

MOTHERFIT2: data will be collected at baseline and 4, 9 and 18 months post-partum.

The case manager fills in a first survey after the inclusion of a participant. For MOTHERFIT1 these questions include, among others, expected delivery date and current medication use. Two weeks after delivery, case managers receive a second survey regarding delivery variables. For MOTHERFIT2 the case manager fills in identical surveys, except the question on expected delivery date.

Participants in the intervention group fill in a training diary and three questions regarding their general physical activity level, weekly. The PPTs will register attendance of the participant during the intervention period and send it by postal mail to the researcher.

### Primary outcome measure

Tables 1 and 2 show the schedule of assessments for MOTHERFIT1 and MOTHERFIT2. The primary outcome measure is self-reported UI based on the severity sum score of the ICIQ-UI-SF. The ICIQ-UI-SF is a brief (four questions) and robust measure for evaluating the frequency of symptoms and impact of UI [34]. The total score ranges from 0 (not affected) to 21 (severely affected). The ICIQ-UI-SF is divided into the following four severity categories: slight (1–5), moderate (6–12), severe (13–18) and very severe (19–21) [35]. The questionnaire is translated in Dutch [36]. Therapy success is defined as absence of UI or change from baseline of at least 3 points on the ICIQ-UI-SF at 18 months post-partum [37].

### Secondary outcome measures

Patient-reported improvement: the Patient Global Impression of Severity (GPE) questionnaire assesses patients’ self-reported improvement [38]. It is an accepted and reliable scale for incontinence, consisting of one question and seven response options [39, 40].

Quality of life outcomes: the Incontinence Impact Questionnaire-7 (IIQ-7) contains seven items that reliably assess the impact of UI on health-related quality of life (HRQL) [41, 42]. It determines UI impact on four domains: mobility, physical functioning, emotional health and embarrassment and ranges from 0 to 100.

General activity level: the diary has to be filled in weekly. Next to a question regarding the number of days PFM exercises have been executed, it contains three questions regarding general activity level. The questions on general activity level are modified from the Dutch healthy exercise norm (Nederlandse Norm Gezond Bewegen). This norm is based on publications of the American College of Sports Medicine [43].

### Adherence to home training programme

Only participants in the intervention group register their performance of requested pelvic-floor-muscle exercises, including their general physical activity, weekly, at home in the training diary. The training diary is a data entry form and, if scanned, an Excel file will be computer generated.

### Cost-effectiveness

For the purpose of the economic evaluation, a study-specific cost questionnaire has been developed. Participants’ resource use ((in) direct costs related to SUI) is collected from the societal and health care perspective. Furthermore, the EuroQol instrument (EQ-5D-5 L) will be administered, a validated, generic health-state measure [43, 44] widely used in economic evaluations. The five-level version (EQ-5D-5 L) is proposed by the recently updated Dutch guideline for economic evaluations in health care [45] and consists of the EQ Visual Analogue Scale and a descriptive system. The descriptive system comprises five dimensions: mobility, self-care, usual activities, pain/discomfort and anxiety/depression. Each dimension can be rated at five levels: no problems, slight problems, moderate problems, severe problems and extreme problems.

### Process evaluation

A study-specific questionnaire has been developed to evaluate patient satisfaction of motherfit group therapy (part 1, 10 items) and satisfaction with the use of the mApp (part 2, seven items). Questions on motherfit group therapy were, e.g. on whether the participant liked training in a group and if there were enough opportunities to ask the motherfit group therapist questions. Questions regarding satisfaction of the mApp were, e.g. on ease of use and whether participants would continue using the mApp after the intervention period. Each item ranges from 1 (strongly disagree) to 5 (strongly agree).

## Statistical methods

### Sample size calculation

Assuming that the average score of the primary outcome measure (ICIQ-UI-SF; range 0–21) of MOTHERFIT1 will lie at 8 and for MOTHERFIT2 at 9 (which is also the expected mean ICIQ-UI-SF score at 18 months post-partum in the CAU group; in contrast, the expected mean ICIQ-UI-SF score at 18 months post-partum in the experimental group is 5 (for MOTHERFIT1 and MOTHERFIT2) together with a relatively high standard deviation of 5 at baseline (because of the non-normality of the measure), participants will – with 97.5% probability – vary at baseline within the ranges of 0 to 19. The minimum acceptable score of participants to be treated is set at 3, so the range lies approximately between 3 and 19. From earlier studies [44], it became clear that the success of the PFMT exercises will be considerable and will be clinically relevant if the gain is higher than half the standard deviation of the baseline, presumably 3 with a somewhat smaller standard deviation of 3, because of the homogenising effects in the experimental arm. In contrast to MOTHERFIT1 (women remain stable), in MOTHERFIT2 it is assumed that the condition of CAU participants at 18 months will worsen with an average ICIQ-UI-SF score going from 9 in the baseline to 10 (SD 5).

Assuming two-sided testing, a power of 90% (beta = 0.10) and a significance level of 0.95 (alpha = 0.05) in each arm of the trial in MOTHERFIT1, minimally 60, and in MOTHERFIT2, minimally 35 participants will have to be included without taking into account that participants may drop out of the study during the 18 months of observations. Using a 20% dropout, in MOTHERFIT1 each arm will need 75 participants, 150 in total, and in MOTHERFIT2 each arm will need 45 participants, 90 in total.

### Statistical analysis

Analysis of the participants will be reported in accordance with the Consolidated Standards of Reporting Trials (CONSORT) Statement [44]. Data will be analysed according to the intention-to-treat principle. By preference, multiple-imputation techniques are used for missing values.

### Descriptive analysis

Firstly, descriptive, univariate statistics will be reported. In case of metric, normally distributed variables, mean and standard deviations are presented. If not normally distributed, medians and percentiles are presented. The Shapiro-Wilk test will be used to assess normality.

Process and structure indicators will be analysed with descriptive statistics and presented as absolute and proportion data (%) whenever the variable is categorical, or as mean (± standard deviation; 95% confidence intervals) or quartiles for continuous variables. A *p* value < 0.05 will be considered to be statistically significant. Data analysis will be carried out using SPSS version 25 (IBM. Corporation, Somers. NY, USA).

### Analysis of main hypotheses

In both studies, the main hypothesis concerns differential changes in ICIQ-UI-SF within time between two randomised groups of participants. (Repeated measurements) analysis of covariance (ANCOVA) will be performed with baseline measurements (T0) as covariate. Transformations of original scores will be attempted if the ICIQ-UI-SF shows a non-normal distribution at T0. Randomisation groups (motherfit group therapy versus CAU) are regarded as a between factor. Next, the within-participants linear trend in time of the outcome will be calculated with the weights from the first orthogonal polynomial contrast and this is used as a dependent variable in a multiple (dummy-)regression analysis. It concerns repeated measurements from T0 to T4 (MOTHERFIT1) and T0 to T3 (MOTHERFIT2). Next to the baseline covariate measurement and the randomisation groups’ dummy variable, other possible confounding variables will be used in this multiple linear regression analysis of the linear trend in time of the ICIQ-UI-SF.

The following potential confounding variables are considered to be used in the multiple linear regression analysis: Body Mass Index (BMI) before pregnancy (> 25), parity, maternal age (> 35 years) and the ability to perform a PFM contraction at baseline.

Forward selection and backward elimination techniques will be used to determine the best fit of the data to a final regression model. Testing of interactive relationships between statistically significant effects of predictors in the final model will be done, especially if it concerns the experimental between-randomisation groups’ factor. Listwise deletion of missing cases will be used in all linear regression modelling. This may be in case of loss-to-follow-up because of a succeeding pregnancy during the follow-up period of 18 months. For the final best-fitting regression model, a residual analysis will be done on the standardised Studentised z-scores and a screening will be performed on outliers to ensure the legitimacy and validity of the use of parametric statistics in analysis by testing the normality of distribution of the linear trend in ICIQ-UI-SF.

Statistical analysis on the secondary outcomes of the study, such as the IIQ-7, the GPE and the EQ-5D-5 L will be handled in the same way as the primary outcome measure ICIQ-UI-SF. Process and structure indicators will be analysed with descriptive statistics and presented quantitatively as numbers and absolute and proportion data.

### Economic evaluation

#### General considerations

For both subgroups in MOTHERFIT1 and MOTHERFIT2, separate trial-based economic evaluations (EE) will be performed, but both EEs will have the same characteristics, except for the time horizon. The EE will take a societal and health care perspective, comparing motherfit group therapy with CAU. The time horizon for MOTHERFIT1 will be (about) 24 months starting from 12 weeks’ gestation (study inclusion) up to 18 months post-partum and for MOTHERFIT2 from approximately 6 weeks to 18 months post-partum. Cost-effectiveness ratios will be expressed as the societal cost per quality-adjusted life-year (QALY) (societal perspective), and the (health care) cost per woman, in whom UI is clinically relevant, reduced (primary outcome; health care perspective). Bootstrap analysis and cost-effectiveness acceptability curves will be constructed, showing for a range of threshold values the probability that motherfit group therapy is cost-effective. Sensitivity analyses and subgroup analyses (e.g. on age categories, adherent versus non-adherent women) will be performed to test for the robustness of the results.

### Cost-analysis

The cost-analysis will be performed from both a societal and health care perspective. Resource use will be measured in natural units and will be valued in monetary terms by multiplying these units by the costs per unit. If available, standardised, national cost prices (e.g. specified by the recently updated Dutch guideline for cost research in health care will be used [46]. Costs are distinguished into motherfit programme costs including the group sessions and home-based part and costs of the mApp (initial and replacement costs for ICT hardware and software), health care costs (e.g. use of incontinence materials, visits to the general practitioner, gynaecologist, midwifery costs, visits to the PPT, surgery, etc.), non-health care costs (e.g. travel costs and productivity losses) and patient and family costs (time spent on the programme, informal care costs). Data on (health care) resource utilisation associated with SUI will be prospectively recorded during the study by the participants. Other health care, non-health care and patient and family costs will be collected by means of a standardised cost questionnaire to be filled out by patients. Costs occurring 12 months after study inclusion will be discounted at 4% according to the Dutch guidelines for economic evaluations health care [47].

### Patient outcome analysis

The outcome for the cost-utility analysis (societal perspective) is defined in terms of QALYs from inclusion up to 18 months post-partum. The number of QALYs is derived from the adjustment of survival data with HRQL. HRQL will be measured with the EuroQol-5D (EQ-5D) instrument, which provides a descriptive health profile and a Dutch valuation set for obtaining utility scores from the EQ-5D [45]. The outcome for the cost-effectiveness analysis (health care perspective) is based on the proportion of women with clinically relevant reduction in UI at 18 months post-partum. Outcomes occurring 12 months following study inclusion will be discounted at 1.5% according to the Dutch guidelines for economic evaluations of health care [47].

### Long-term decision analytical modelling

Next to the trial-based EE, a model-based EE will be performed, as it is expected that the economic impact of motherfit is best investigated by means of a long-term decision analytical model. First a structure and working model will be created that will facilitate the necessary analysis to be performed throughout the project. This model will be able to incorporate the values of all input parameters (both point estimates and uncertainty). Once the structure of the model is established, four essential types of data will be required: probabilities, costs, survival and health utilities (QALYs). Short-term costs and effectiveness data are readily available from the trial-based EE, whereas longer-term data may require synthesis of available evidence in the literature. Estimates of the economic impact will first be made using fixed estimates of probabilities, costs and health outcomes. Subsequently, a probabilistic sensitivity analysis will be performed which will address the joint uncertainty of the model inputs. As for the trial-based cost-effectiveness analysis, cost-effectiveness acceptability curves will be constructed. As with the trial-based EE, the model-based EE will address the cost per QALY (societal perspective) and cost per UI prevented (health care perspective). We will express uncertainty by means of confidence intervals and by creating cost-effectiveness acceptability curves. The appropriate time horizon will be agreed upon during the study but is expected to be lifetime.

### Budget Impact Analysis (BIA)

A BIA will be performed according to the International Society for Pharmacoeconomics and Outcomes Research (ISPOR) guidelines [48]. The BIA addresses the financial stream of consequences related to the implementation of motherfit group therapy and thus its affordability. The budget impact will depend, e.g. on patient acceptability of the programme, the uptake of the programme by health care professional and the target group, the cost-increase due to increased implementation of motherfit group therapy, and the cost savings due to preventing or reducing UI, i.e. reduced cost-of-illness. The structure and some data input of the decision analytical model developed for the EE will be adapted for the BIA. Input parameters will be based on results of the trial, national prevalence data, unit prices and tariffs obtained in the trial-based EE, and from the available literature when necessary. The analyses will be performed from different perspectives, including a health care budgetary perspective and a health insurers’ perspective. The model will take changes in the adoption/implementation of the programme, and patient acceptability/uptake into account and will compare different scenarios as regards to the swiftness and extensiveness of the uptake. In order to test the robustness of the results, sensitivity analyses will be performed. The time horizon will be varied from 1 year up to 5 years. No discounting will be applied.

### Withdrawal of individual subjects

Subjects can leave the study at any time and for any reason, if they wish to do so, without any consequences. The investigator can decide to withdraw a subject from the study for urgent medical reasons.

All women enrolled in the study will be followed and accounted for. Women who are unwilling or unable to commit themselves to the study plan and follow-up schedule (i.e. serious illness, during pregnancy, e.g. premature rupture of membranes, blood loss, severe high blood pressure, pre-eclampsia, movement out of the local area, etc.) may be withdrawn from the study. Women who will become pregnant again during the follow-up period of 18 months will be handled as dropout cases. Upon withdrawal of a subject, all documentation is available immediately for the investigators through the electronic case report file.

## Monitoring

This will be by the Clinical Trial Center Maastricht (CTCM), Christel Jacquot, Oxfordlaan 70, 6229 EV Maastricht, The Netherlands (www.ctcm.nl).

## Discussion

The two motherfit studies are studies aim to evaluate whether motherfit group therapy is (cost-)effective 18 months post-partum for pregnant (MOTHERFIT1) and post-partum women (MOTHERFIT2) with SUI. As health care costs are rising in general, there is a need for cost-effective strategies, which is one of the main reasons for initiating the motherfit studies. The motherfit studies are, to our knowledge, the first studies that evaluate both longer-term results and health care costs compared to CAU in pregnant and post-partum women with SUI. The endpoint of 18 months post-partum is chosen because of the increasing possibility of a subsequent pregnancy and consequently loss to follow-up. In order to sustain long-term results, it is known that adherence is a strong predictive factor [33]. Therefore, motherfit group therapy not only focusses on PFMT, general fitness exercises and education, but also has a strong emphasis on adherence and self-management. Adherence to PFMT will be supported by a mApp.

Currently, no guidelines on urinary incontinence exist specifically for pregnant and post-partum women. In case motherfit demonstrates to be (cost-)effective, implementation of motherfit group therapy should be considered in peri-partum care and future guidelines.

## Trial status

Participants for the MOTHERFIT1 and MOTHERFIT2 studies are currently being recruited in five regions in the southern part of The Netherlands. The first patient was randomised on 4 October 2017. Last participant follow-up for MOTHERFIT1 and MOTHERFIT2 is expected in August 2020; version 5, 31 March 2017. The trial registration number is NTR5971 (Dutch Trial Register), 18 July 2016.

## Additional file

Additional file 1: Standard Protocol Items: Recommendations for Interventional Trials (SPIRIT Checklist. (DOCX 139 kb)

## Abbreviations

BIA: Budget Impact Analysis
CAU: Care-as-usual
CTCM: Clinical Trial Center Maastricht,
EE: Economic evaluation
GEE: Patient Global Impression of Severity
ICIQ-UI-SF: International Consultation on Incontinence Questionnaire-Short Form
IIQ-7: Incontinence Impact Questionnaire
ISPOR: International Society For Pharmacoeconomics and Outcomes Research
LOCF: Last observation carried forward
mApp: Mobile app
NVOQ-q: Nederlandse Vereniging voor Obstetrie & Gynaecologie vragenlijst
PFM(T): Pelvic-floor-muscle (training)
PFMGT: Pelvic-floor-muscle group therapy
PPT: Pelvic physiotherapist
QALY: Quality-adjusted life-year
QoL: Quality of life
RCT: Randomised controlled trial
(S)UI: (Stress) urinary incontinence
WHO: World Health Organisation

## References

[1] Altaweel W, Alharbi M (2012). Urinary incontinence: prevalence, risk factors, and impact on health related quality of life in Saudi women. Neurourol Urodyn.

[2] Cooper J, Annappa M, Quigley A, Dracocardos D, Bondili A, Mallen C (2015). Prevalence of female urinary incontinence and its impact on quality of life in a cluster population in the United Kingdom (UK): a community survey. Prim Health Care Res Dev.

[3] Irwin DE, Milsom I, Hunskaar S, Reilly K, Kopp Z, Herschorn S, Coyne K, Kelleher C, Hampel C, Artibani W, Abrams P (2006). Population-based survey of urinary incontinence, overactive bladder, and other lower urinary tract symptoms in five countries: results of the EPIC study. Eur Urol.

[4] Andersson G, Johansson JE, Garpenholt O, Nilsson K (2004). Urinary incontinence—prevalence, impact on daily living and desire for treatment: a population-based study. Scand J Urol Nephrol.

[5] Delancey JO (2010). Why do women have stress urinary incontinence?. Neurourol Urodyn.

[6] Haylen BT, de Ridder D, Freeman RM, Swift SE, Berghmans B, Lee J, Monga A, Petri E, Rizk DE, Sand PK, Schaer GN (2010). An International Urogynecological Association (IUGA)/International Continence Society (ICS) joint report on the terminology for female pelvic floor dysfunction. Int Urogynecol J.

[7] Delancey JO, Ashton-Miller JA (2004). Pathophysiology of adult urinary incontinence. Gastroenterology.

[8] Chan SS, Cheung RY, Yiu KW, Lee LL, Chung TK (2013). Prevalence of urinary and fecal incontinence in Chinese women during and after their first pregnancy. Int Urogynecol J.

[9] Solans-Domenech M, Sanchez E, Espuna-Pons M (2010). Urinary and anal incontinence during pregnancy and postpartum: incidence, severity, and risk factors. Obstet Gynecol.

[10] Morkved S, Bo K (1999). Prevalence of urinary incontinence during pregnancy and postpartum. Int Urogynecol J Pelvic Floor Dysfunct.

[11] Brown S, Gartland D, Perlen S, McDonald E, MacArthur C (2015). Consultation about urinary and faecal incontinence in the year after childbirth: a cohort study. BJOG.

[12] Buurman MB, Lagro-Janssen AL (2013). Women’s perception of postpartum pelvic floor dysfunction and their help-seeking behaviour: a qualitative interview study. Scand J Caring Sci.

[13] MacArthur C, Wilson D, Herbison P, Lancashire RJ, Hagen S, Toozs-Hobson P, Dean N, Glazener C (2016). Urinary incontinence persisting after childbirth: extent, delivery history, and effects in a 12-year longitudinal cohort study. BJOG.

[14] Viktrup L, Lose G (2001). The risk of stress incontinence 5 years after first delivery. Am J Obstet Gynecol.

[15] Hermansen IL, O’Connell BO, Gaskin CJ (2010). Women’s explanations for urinary incontinence, their management strategies, and their quality of life during the postpartum period. J Wound Ostomy Continence Nurs.

[16] Handa VL, Zyczynski HM, Burgio KL, Fitzgerald MP, Borello-France D, Janz NK, Fine PM, Whitehead W, Brown MB, Weber AM (2007). The impact of fecal and urinary incontinence on quality of life 6 months after childbirth. Am J Obstet Gynecol.

[17] Hagglund D, Walker-Engstrom ML, Larsson G, Leppert J (2003). Reasons why women with long-term urinary incontinence do not seek professional help: a cross-sectional population-based cohort study. Int Urogynecol J Pelvic Floor Dysfunct.

[18] Hagglund D, Wadensten B (2007). Fear of humiliation inhibits women’s care-seeking behaviour for long-term urinary incontinence. Scand J Caring Sci.

[19] Miller JM, Sampselle C, Ashton-Miller J, Hong GR, DeLancey JO (2008). Clarification and confirmation of the Knack maneuver: the effect of volitional pelvic floor muscle contraction to preempt expected stress incontinence. Int Urogynecol J Pelvic Floor Dysfunct.

[20] Boyle R, Hay-Smith EJ, Cody JD, Morkved S (2012). Pelvic floor muscle training for prevention and treatment of urinary and faecal incontinence in antenatal and postnatal women. Cochrane Database Syst Rev.

[21] Albers-Heitner P, Berghmans B, Nieman F, Lagro-Janssen T, Winkens R (2008). Adherence to professional guidelines for patients with urinary incontinence by general practitioners: a cross-sectional study. J Eval Clin Pract.

[22] van Gerwen MA, Schellevis FG, Lagro-Janssen AL (2009). Management of urinary incontinence in general practice: data from the Second Dutch National Survey. J Eval Clin Pract.

[23] Paiva LL, Ferla L, Darski C, Catarino BM, Ramos JG (2017). Pelvic floor muscle training in groups versus individual or home treatment of women with urinary incontinence: systematic review and meta-analysis. Int Urogynecol J.

[24] CBS StatLine. Zorguitgaven. https://www.cbs.nl/nl-nl/nieuws/2017/20/zorguitgaven-stijgen-in-2016-met-1-8-procent. Accessed 19 Sept 2018.

[25] Lamb SE, Pepper J, Lall R, Jorstad-Stein EC, Clark MD, Hill L, Fereday-Smith J (2009). Group treatments for sensitive health care problems: a randomised controlled trial of group versus individual physiotherapy sessions for female urinary incontinence. BMC Womens Health.

[26] Dumoulin C, Alewijnse D, Bo K, Hagen S, Stark D, Van Kampen M, Herbert J, Hay-Smith J, Frawley H, McClurg D, Dean S (2015). Pelvic-floor-muscle training adherence: tools, measurements and strategies-2011 ICS State-of-the-Science Seminar Research Paper II of IV. Neurourol Urodyn.

[27] Timmermans L, Falez F, Melot C, Wespes E (2013). Validation of use of the International Consultation on Incontinence Questionnaire-Urinary Incontinence-Short Form (ICIQ-UI-SF) for impairment rating: a transversal retrospective study of 120 patients. Neurourol Urodyn.

[28] Ministerie van Volksgezondheid Welzijn en Sport (2014). Medisch-wetenschappelijk onderzoek; algemene informatie voor de proefpersoon.

[29] Slieker-ten Hove MC, Pool-Goudzwaard AL, Eijkemans MJ, Steegers-Theunissen RP, Burger CW, Vierhout ME (2009). The prevalence of pelvic organ prolapse symptoms and signs and their relation with bladder and bowel disorders in a general female population. Int Urogynecol J Pelvic Floor Dysfunct.

[30] Westerik-Verschuuren L, Moossdorff-Steinhauser H. Beroepsprofiel bekkenfysiotherapeut: Royal Dutch Society for Physical Therapy (KNGF); 2014. https://www.kngf.nl/binaries/content/assets/kngf/onbeveiligd/vakgebied/vakinhoud/beroepsprofielen/beroepsprofiel-bekkenfysiotherapeut.pdf. Accessed 5 Nov 2018.

[31] Bo K, Talseth T, Holme I (1999). Single blind, randomised controlled trial of pelvic floor exercises, electrical stimulation, vaginal cones, and no treatment in management of genuine stress incontinence in women. BMJ.

[32] Haskell WL, Lee IM, Pate RR, Powell KE, Blair SN, Franklin BA, Macera CA, Heath GW, Thompson PD, Bauman A (2007). Physical activity and public health: updated recommendation for adults from the American College of Sports Medicine and the American Heart Association. Med Sci Sports Exerc.

[33] Latorre GFS, de Fraga R, Seleme MR, Mueller CV, Berghmans B. An ideal e-health system for pelvic floor muscle training adherence: systematic review. Neurourol Urodyn. 2018;38(1):63–80. 10.1002/nau.23835. Epub 2018 Oct 30. 10.1002/nau.23835 30375056

[34] Avery K, Donovan J, Peters TJ, Shaw C, Gotoh M, Abrams P (2004). ICIQ: a brief and robust measure for evaluating the symptoms and impact of urinary incontinence. Neurourol Urodyn.

[35] Klovning A, Avery K, Sandvik H, Hunskaar S (2009). Comparison of two questionnaires for assessing the severity of urinary incontinence: the ICIQ-UI SF versus the Incontinence Severity Index. Neurourol Urodyn.

[36] Bristol Urological Institute (2014). International Consultation on Incontinence Modular Questionnaire (ICIQ). ICIQ Structure Short Form.

[37] Nystrom E, Sjostrom M, Stenlund H, Samuelsson E (2015). ICIQ symptom and quality of life instruments measure clinically relevant improvements in women with stress urinary incontinence. Neurourol Urodyn.

[38] Hudak PL, Wright JG (2000). The characteristics of patient satisfaction measures. Spine (Phila PA 1976).

[39] Kamper SJ, Ostelo RW, Knol DL, Maher CG, de Vet HC, Hancock MJ (2010). Global Perceived Effect scales provided reliable assessments of health transition in people with musculoskeletal disorders, but ratings are strongly influenced by current status. J Clin Epidemiol.

[40] Yalcin I, Bump RC (2003). Validation of two global impression questionnaires for incontinence. Am J Obstet Gynecol.

[41] Uebersax JS, Wyman JF, Shumaker SA, McClish DK, Fantl JA (1995). Short forms to assess life quality and symptom distress for urinary incontinence in women: the Incontinence Impact Questionnaire and the Urogenital Distress Inventory. Continence Program for Women Research Group. Neurourol Urodyn.

[42] van der Vaart CH, de Leeuw JR, Roovers JP, Heintz AP (2003). Measuring health-related quality of life in women with urogenital dysfunction: the urogenital distress inventory and incontinence impact questionnaire revisited. Neurourol Urodyn.

[43] Garber CE, Blissmer B, Deschenes MR, Franklin BA, Lamonte MJ, Lee IM, Nieman DC, Swain DP (2011). American College of Sports Medicine position stand. Quantity and quality of exercise for developing and maintaining cardiorespiratory, musculoskeletal, and neuromotor fitness in apparently healthy adults: guidance for prescribing exercise. Med Sci Sports Exerc.

[44] Hay-Smith EJ, Herderschee R, Dumoulin C, Herbison GP. Comparisons of approaches to pelvic floor muscle training for urinary incontinence in women. Cochrane Database Syst Rev. 2011:Cd009508. 10.1002/14651858.CD009508. 10.1002/14651858.CD009508 22161451

[45] Versteegh M, Vermeulen K, Evers S, de Wit G, Prenger R, Stock E (2016). Dutch tariff for the five-level version of EQ-5D. Value Health.

[46] Zorginstituut Nederland. Methodologie van kostenonderzoek en referentieprijzen voor economische evaluaties in de gezondheidszorg; 2015. https://www.zorginstituutnederland.nl/publicaties/publicatie/2016/02/29/richtlijn-voor-het-uitvoeren-van-economische-evaluaties-in-de-gezondheidszorg. Accessed 19 Sept 2018.

[47] Zorginstituut Nederland. Richtlijn voor het uitvoeren van economische evaluaties in de gezondheidszorg. Nederland; 2015. p. 1.

[48] Sullivan SD, Mauskopf JA, Augustovski F, Jaime Caro J, Lee KM, Minchin M, Orlewska E, Penna P, Rodriguez Barrios JM, Shau WY (2014). Budget impact analysis—principles of good practice: report of the ISPOR 2012 Budget Impact Analysis Good Practice II Task Force. Value Health.

